# Supplementary material for: The psychological correlates of distinct neural states occurring during wakeful rest
Source: Sci Rep. 2020 Dec 3;10:21121. doi: 10.1038/s41598-020-77336-z (PMC7712889; doi:10.1038/s41598-020-77336-z)
Supplement: Supplementary file 1 — Supplementary Materials [file 41598_2020_77336_MOESM1_ESM.pdf]

Supplementary Information for

The psychological correlates of distinct neural states occurring during wakeful rest

Theodoros Karapanagiotidis, Diego Vidaurre, Andrew J. Quinn, Deniz Vatansever, Giulia L. Poerio, Adam Turnbull, Nerissa Siu Ping Ho, Robert Leech, Boris C. Bernhardt, Elizabeth Jefferies, Daniel S. Margulies, Thomas E. Nichols, Mark W. Woolrich, Jonathan Smallwood

Theodoros Karapanagiotidis.
E-mail: theodoros.karapanagiotidis@york.ac.uk

This PDF file includes:

Supplementary text
Figures S1 to S11
Tables S1 to S3
References for SI citations

Supporting Information Text

SI Materials and Methods

Experience sampling questions. We asked participants to answer 25 questions shown in Table S1 at the end of the resting state functional Magnetic Resonance Imaging (rs-fMRI) scan, relating to their thoughts during this period. Answers were given on a 4-scale Likert scale ranging from "Not at all" to "Completely".

Physical and mental health questionnaires. Quality of life, physical and psychological health, social relationships and environmental well-being were measured by the World Health Organization Quality of Life WHOQOL-BREF instrument[1]. Private and public self-consciousness and social anxiety were assessed using the Self-Consciousness scale[2], state and trait anxiety by the State-Trait Anxiety inventory[3] and trait rumination by the Ruminative response scale[4]. Finally, depression was measured using the CES-D scale[5], autism by the Autism Spectrum Quotient[6] and ADHD by the World Health Organization Adult ADHD Self-Report scale[7].

SI Results

HMM stability. To evaluate the stability of our HMM decompositions, we ran the algorithm 10 times and investigated the similarity of the models. Similarity was measured as the correlation between the state time series after optimally reordering the states of each model using the Munkres assignment algorithm[8]. We found that the 7-state solution produced the same decomposition across all 10 iterations. We repeated the procedure for number of states s = 9 and found that the stability of the solutions was reduced (average similarity $\mu_9 = 0.80$). We then divided our sample randomly into two halves and found that the 7-state solutions were again relatively more reliable (7-states: $\mu_{7split1} = 0.90$ and $\mu_{7split2} = 0.94$, 9-states: $\mu_{9split1} = 0.71$ and $\mu_{9split2} = 0.80$, Fig. S2).

9-state HMM dwell-times and relation to behaviour. We performed the same analyses aimed at identifying how the mean dwell-time of the dynamic states varied with measures of ongoing experience and trait measures of well-being for an HMM decomposition of 9 states. These analyses revealed that the dwell-time of states 1 and 5 was associated with differential patterns of reports made by our participants at the end of the scan ($F(8,236) = 2.14$, $p = 0.033$, Wilks' $\Lambda = 0.933$, partial $\eta^2 = 0.067$ for state 1 and $F(8,236) = 2.1$, $p = 0.037$, Wilks' $\Lambda = 0.934$, partial $\eta^2 = 0.066$ for state 5 accordingly). We also found that three of the states had a multivariate association with trait measures of well-being (state 1, $F(3,153) = 3.16$, $p = 0.027$, Wilks' $\Lambda = 0.942$, partial $\eta^2 = 0.058$, state 6, $F(3,153) = 4.66$, $p = 0.004$, Wilks' $\Lambda = 0.916$, partial $\eta^2 = 0.084$, and state 8, $F(3,153) = 2.77$, $p = 0.044$, Wilks' $\Lambda = 0.948$, partial $\eta^2 = 0.052$). These results are presented in Figure S6.

7-state HMM following global signal regression. We ran the same analysis pipeline following global signal regression (GSR) for an HMM decomposition of 7 states. The HMM decompositions were stable and relatively similar to the 7-state ones where no GSR was performed ($r = 0.59$, similarity was assessed in the same way as before). Figure S4 shows the spatial similarity between the states' maps from the decompositions with and without GSR. We then performed the same multi-variate analyses, including motion, age and gender as co-variates of no interest. These analyses revealed that the dwell-time of state 6 was associated with differential patterns of reports made by our participants at the end of the scan ($F(8,238) = 2.09$, $p = 0.038$, Wilks' $\Lambda = 0.934$, partial $\eta^2 = 0.066$). We also found that state 5 had a multivariate association with trait measures of well-being ($F(3,155) = 3.58$, $p = 0.015$, Wilks' $\Lambda = 0.935$, partial $\eta^2 = 0.065$). These results are presented in Figure S7. State 6 shows a pattern of neural activity often seen during tasks; it is most similar to state 3 from our main analysis (see Fig. S4) and shows a similar association to ongoing experience. Participants who spent longer on this state reported fewer spontaneous thoughts, focused on distant time problem solving. In the case of well-being, spending more time in state 5 was linked to increased anxiety, rumination, and self-consciousness. This state did not have an explicit spatially homologue state from the original decomposition, but rather resembled a mixture of some of the original states. However, its correlates to well-being were similar across individuals to those of state 7 from our main analysis (Fig. S9), which had links to similar traits associated with negative affect. In addition, although we did not find a similar link to experience like the one of state 7 from the no GSR HMMs with reports of intrusive thoughts about the past, this pattern was most similar to that of states 5 and 6 of the HMM after GSR. Finally, Figure S11 shows that when examining the spatial similarity of the states with the three neuro-cognitive hierarchies from[9], we found the same pattern as in our other analyses, where the synthetic states clustered towards the middle of the three dimensions, and the states generated based on real data fell, on average, away from the centre and towards the outer edge of the distribution of synthetic states.

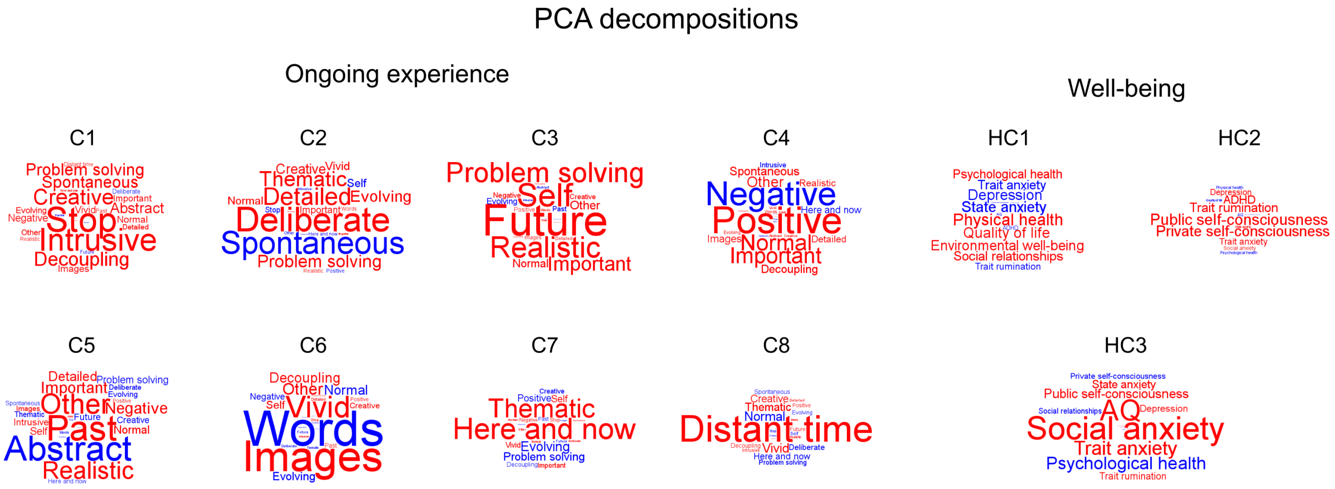

Figure S1. *Behavioural variables.* Component weights from principal component analyses on the scores describing the participants' experience at the end of the resting state scan and trait measures of well-being, visualised using a word cloud. Font size represents the strength of the association and font colour its sign (red for positive and blue for negative values).

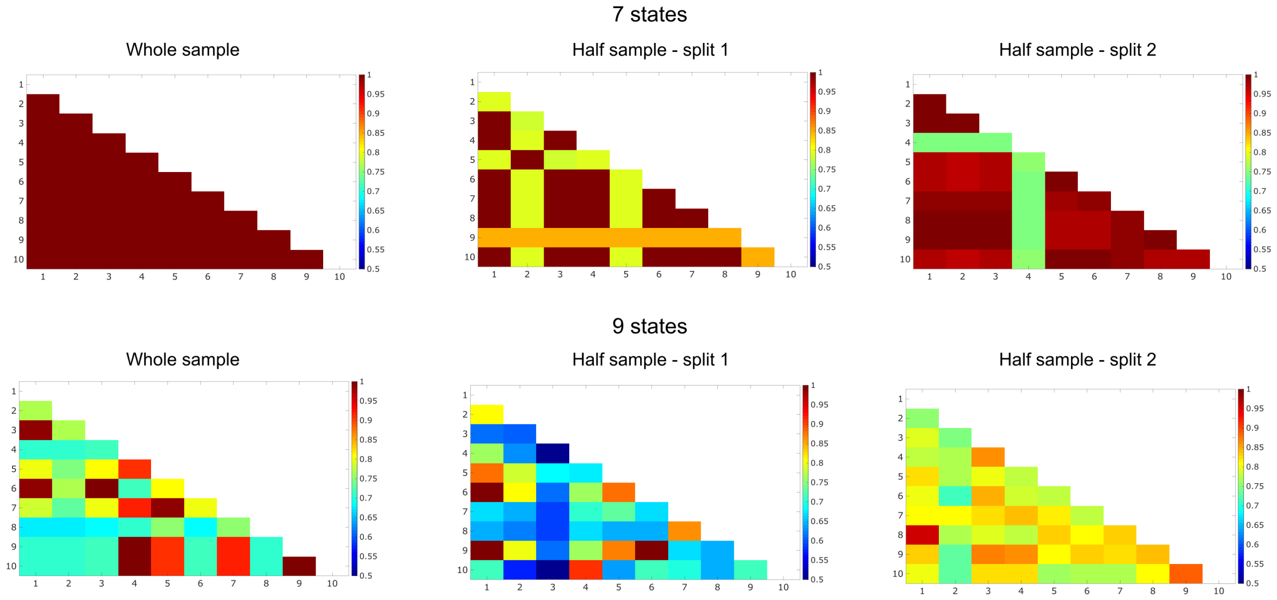

Figure S2. *Hidden Markov models similarities*. Similarities between the hidden Markov models after multiple runs of the algorithm for 7 and 9 states using the whole sample and split-halves.

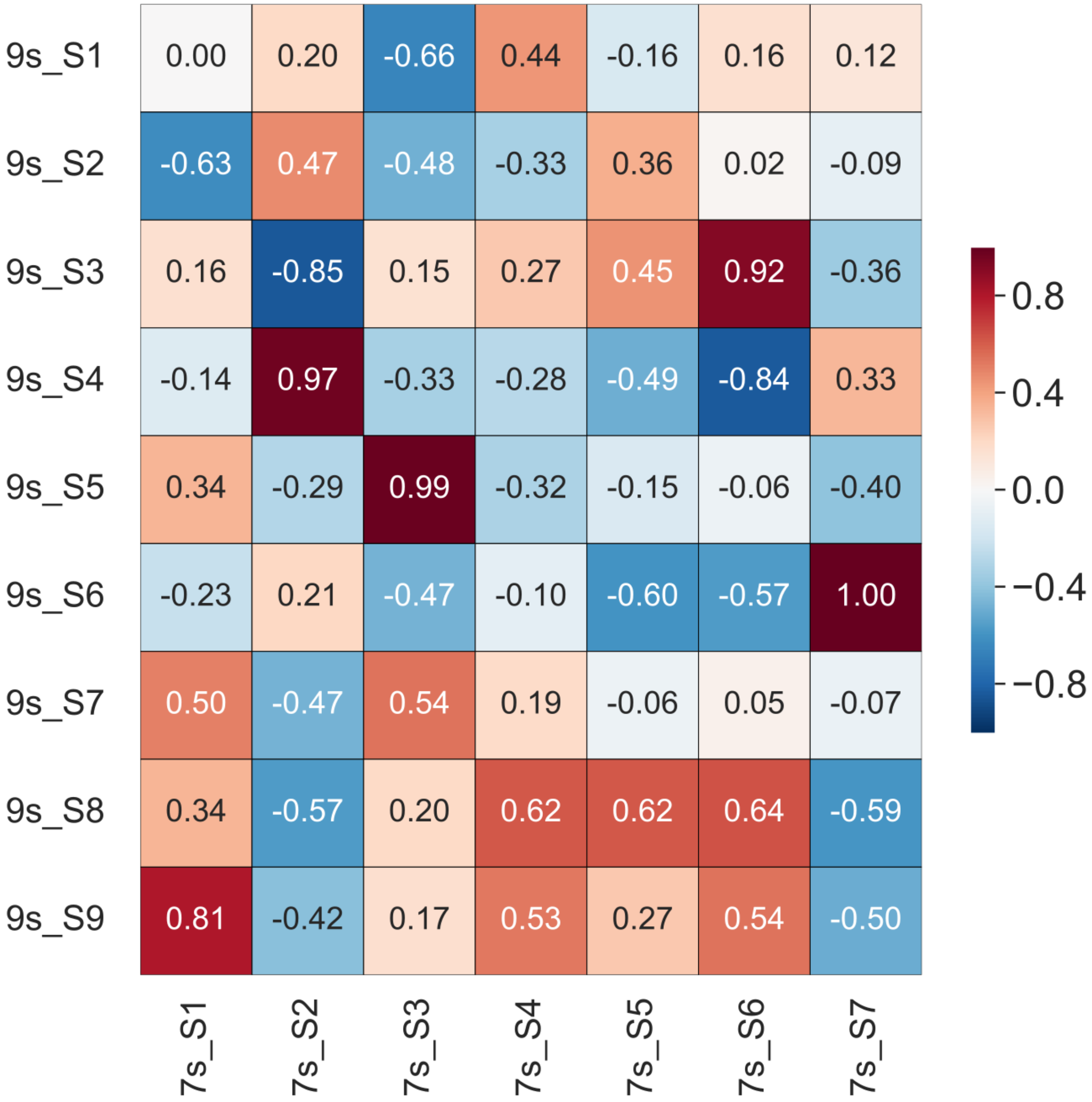

Figure S3. *Hidden Markov model states' spatial maps similarities*. Pair-wise correlations between the state spatial maps from an HMM decomposition of our data for 7 and 9 states.

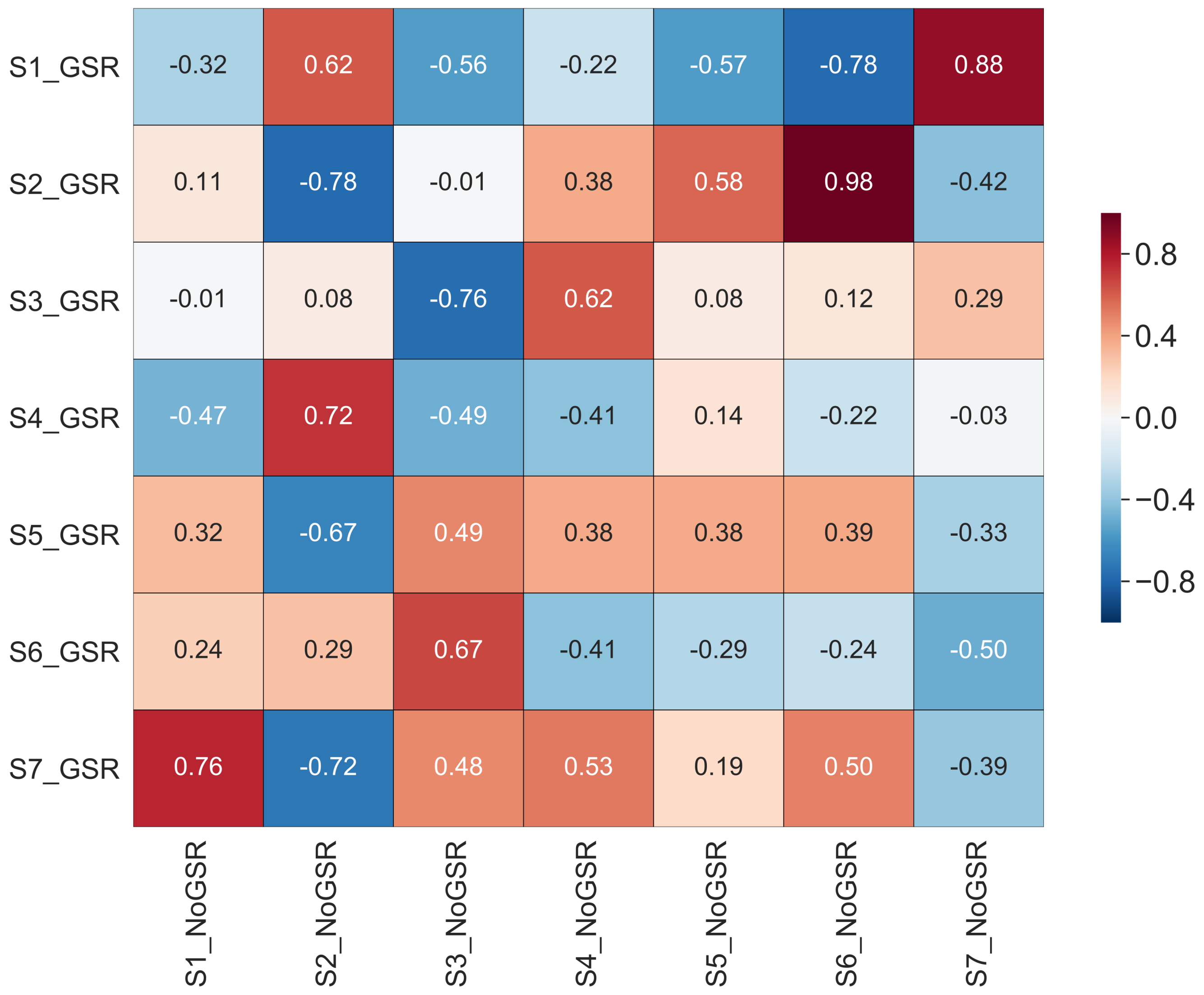

Figure S4. *Hidden Markov model states' spatial maps similarities*. Pair-wise correlations between the state spatial maps from an HMM decomposition of our data for 7 states with and without GSR performed.

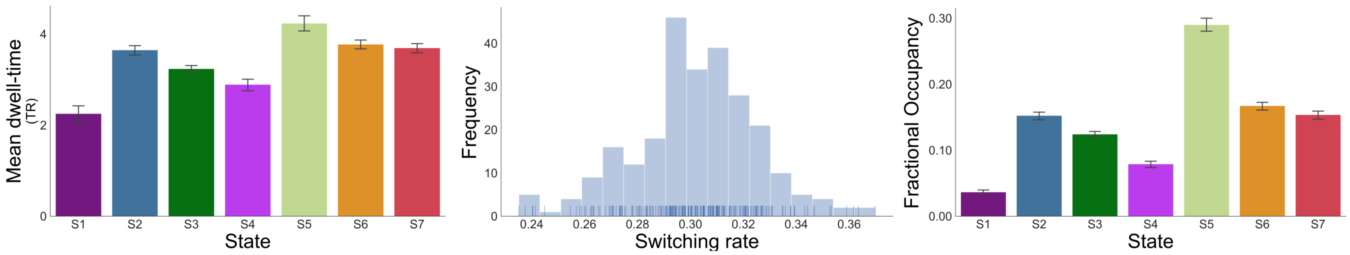

Figure S5. *HMM summary metrics*. **Left**. Bar plot of the mean dwell-time of each state (± 95% confidence intervals). **Middle**. Histogram of subjects' switching rate between states. **Right**. Bar plot of the average time spend on each state (fractional occupancy) (± 95% confidence intervals).

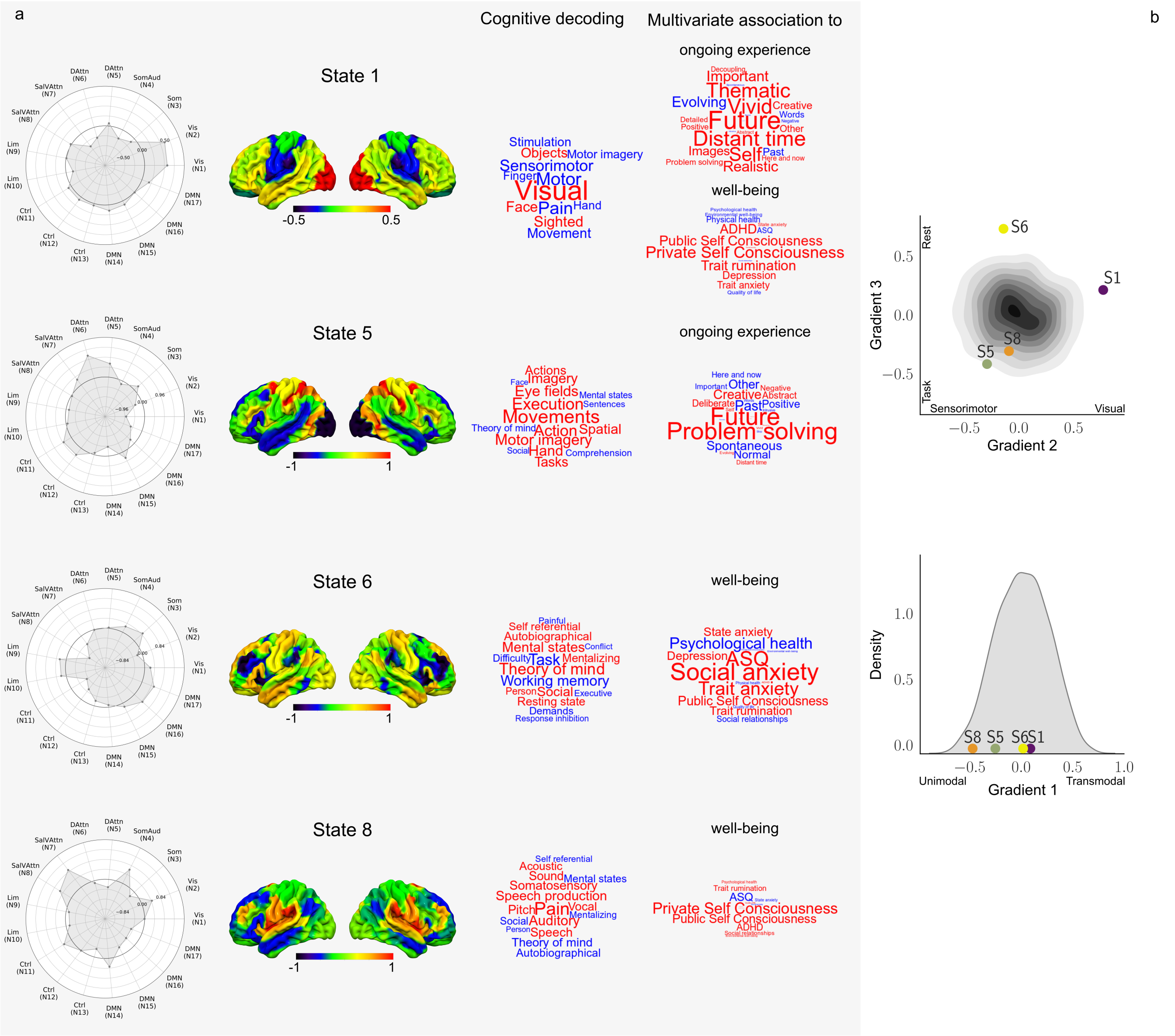

Figure S6. *Associations between the mean dwell-time of neural states with experience and well-being, and their relationship to neurocognitive hierarchies, for the 9-states HMM decomposition*. **a**) This panel shows the spatial distribution of the states as well as a meta-analysis of these spatial maps using Neurosynth (cognitive decoding) which is displayed in the form of word clouds. The word clouds on the right show the pattern of answers associated with each state. Font size represents the strength of the association and font colour its sign (red for positive and blue for negative values). In addition, we show for each state, the relative contributions of each of the networks used in the HMM analyses, in the form of radar plots. **b**)This panel shows the spatial similarity between the spatial maps describing three large-scale neurocognitive hierarchies[9] and the spatial maps generated from the real data. N1-N17: Network 1 to network 17 from the Yeo network parcellation[10]. Vis: Visual, Som: Somatomotor, SomAud: Somatomotor/Auditory, DAttn: Dorsal attention, SalVAttn: Salience/Ventral attention, Lim: Limbic, Ctrl: Control, DMN: Default.

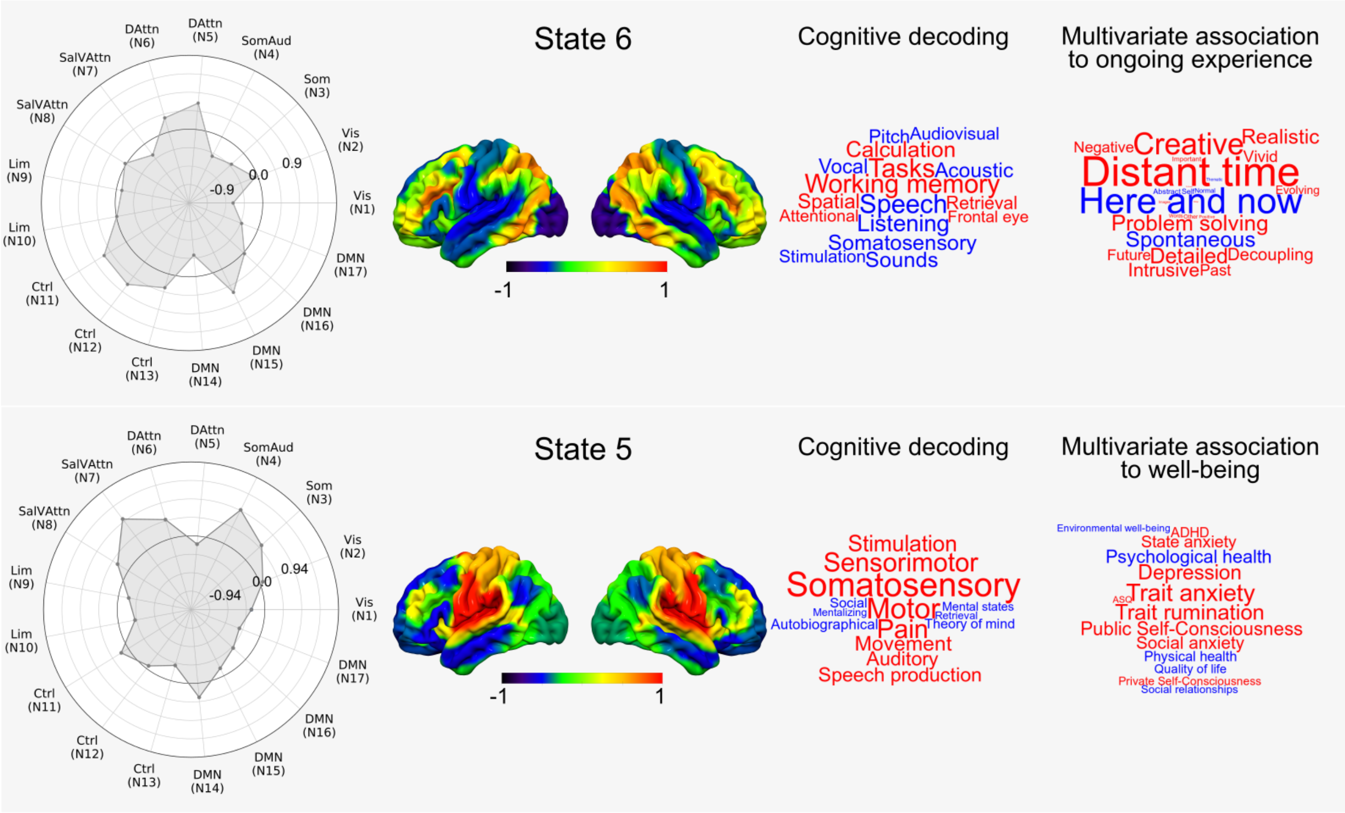

Figure S7. *Associations between the mean dwell-time of neural states with experience and well-being for the 7-states HMM decomposition following GSR*. This figure shows the spatial distribution of the two states as well as a meta-analysis of these spatial maps using Neurosynth (cognitive decoding), which are displayed in the form of word clouds. The word clouds on the right show the pattern of answers associated with each state. In addition, we show, for each state, the relative contributions of each of the networks used in the HMM analyses, in the form of radar plots. N1-N17: Network 1 to network 17 from the Yeo network parcellation[10]. Vis: Visual, Som: Somatomotor, SomAud: Somatomotor/Auditory, DAttn: Dorsal attention, SalVAttn: Salience/Ventral attention, Lim: Limbic, Ctrl: Control, DMN: Default.

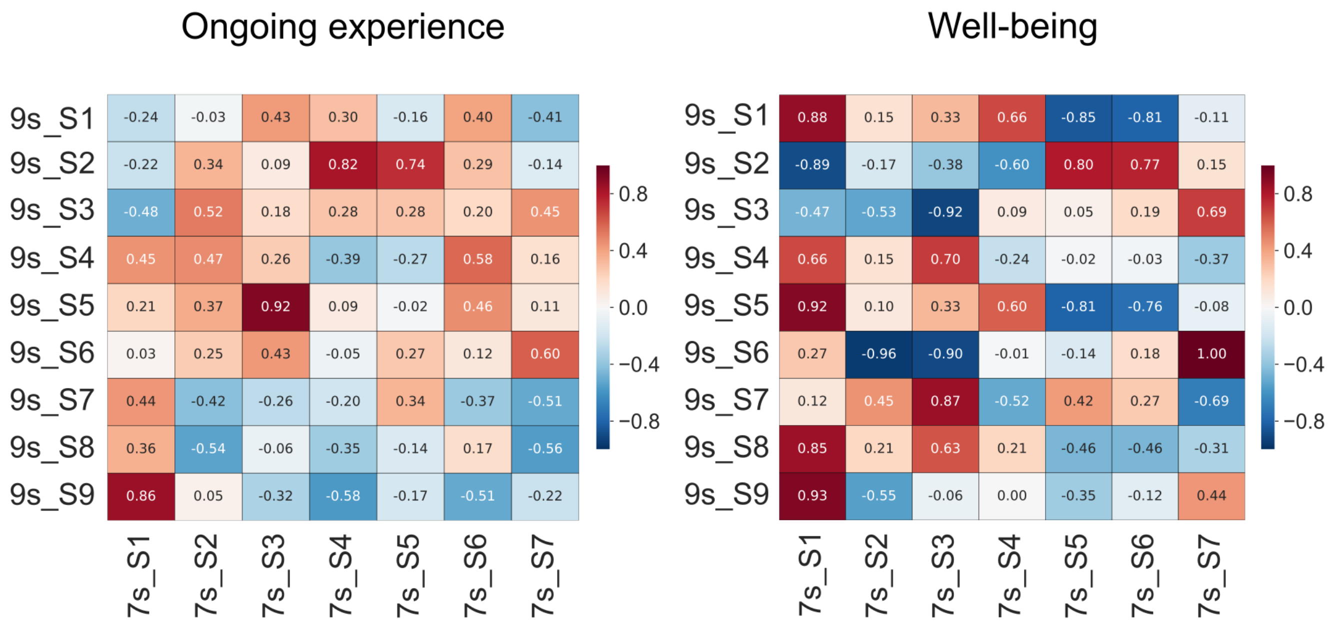

Figure S8. *Similarities between the 7-state and 9-state dwell-time experiential correlates across individuals.* Heat maps showing the correlations between the multivariate patterns of experience and well-being associated with the states' mean dwell-time from the 7-state and 9-state solutions across individuals.

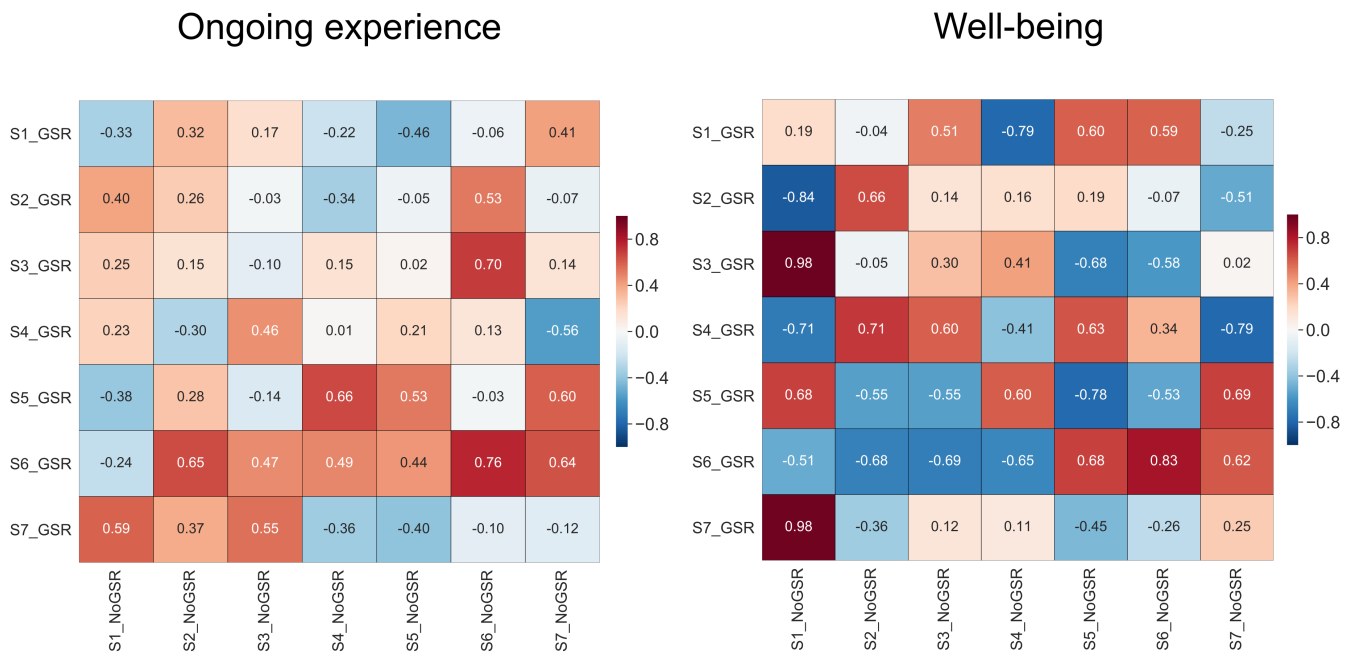

Figure S9. *Similarities between the 7-state dwell-time experiential correlates across individuals from the analyses with and without GSR*. Heat maps showing the correlations between the multivariate patterns of experience and well-being associated with the states' mean dwell-time from the 7-state solutions with and without GSR across individuals.

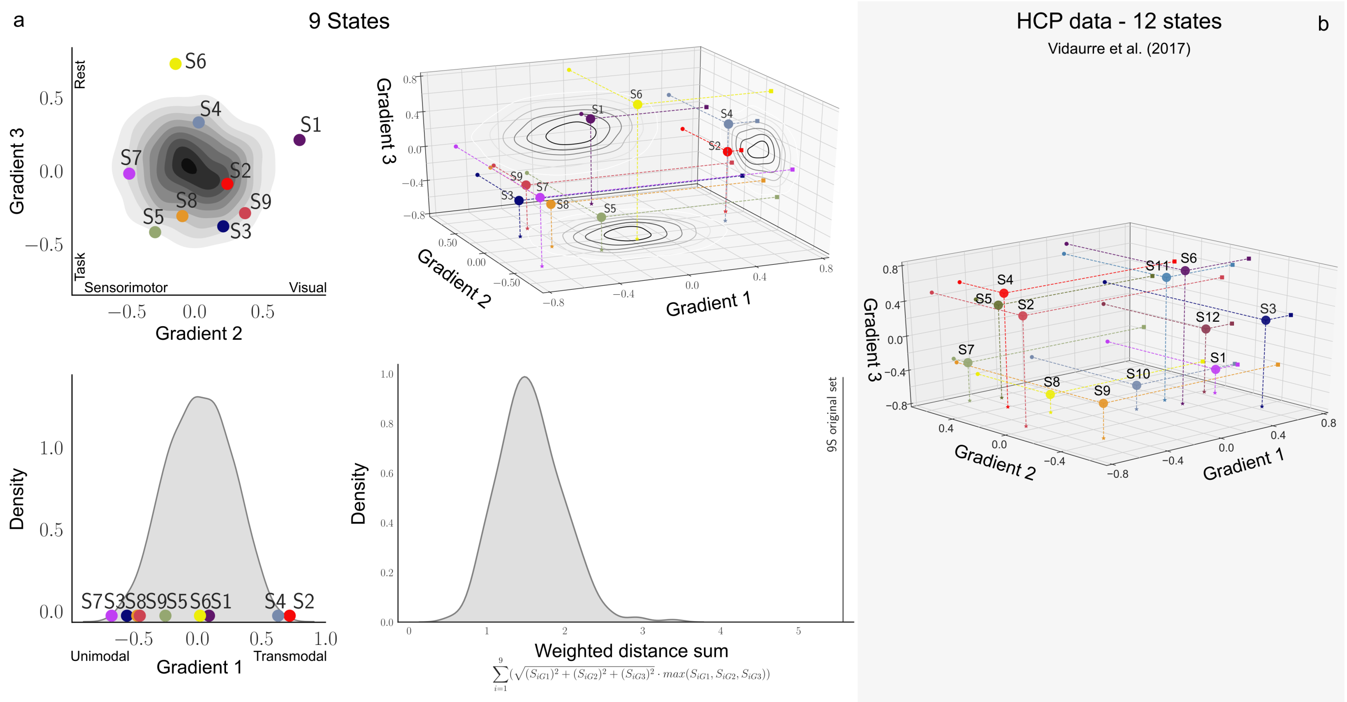

Figure S10. *Relationship between naturally occurring states at rest and three well-established cognitive hierarchies, for different HMM decompositions*. **a**) This panel shows the spatial similarity between the spatial maps describing three large-scale neurocognitive hierarchies[9] and the set of spatial maps generated from a 9-states HMM decomposition of the real data. In these figures the contour plots projected onto each plane describe the distribution of values generated through permutation. The lower right-hand histogram shows the topology for each set of states generated synthetically as well as for the real data, represented as the sum of the weighted distance from the origin of each point in each set. **b**) This panel shows a three-dimensional scatter plot of the similarities between the functional gradient spatial maps and 12 states inferred from running an HMM on the Human Connectome Project data[11].

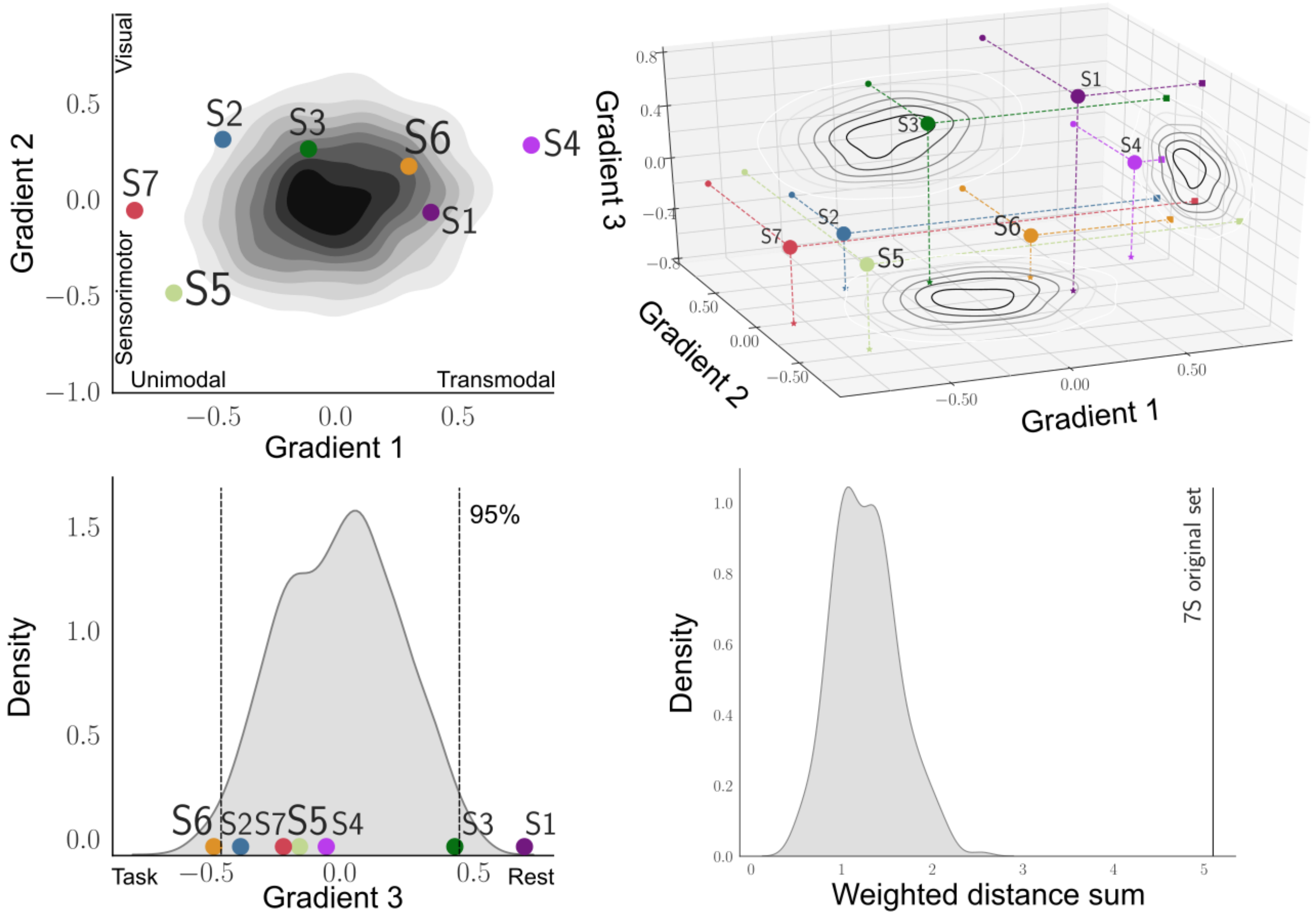

Figure S11. *Relationship between naturally occurring states at rest and three well-established cognitive hierarchies following GSR*. This figure shows the spatial similarity between the spatial maps describing three neural hierarchies identified by[9], and the set of spatial maps generated from the HMM decomposition of the real data following GSR. In these figures, the contour plots projected onto each plane describe the distribution of the synthetic states generated through permutation. The upper and lower left plots show where each empirical state falls on the gradient 1-2 plane, and on the gradient 3 axis respectively. The area between the dotted lines in the density plot shows where 95% of the synthetic states fall on the gradient 3 axis. The lower right-hand histogram shows how far away the states are from the origin of gradient space as a set. This is calculated as the sum of the weighted distance from the origin of each point in each set. The line shows where the set of the empirical states fall, whereas the shaded histogram shows the distribution of the synthetic states sets. States 5, and 6, whose dwell-time was associated with psychological measures, are highlighted.

Table S1. Experience sampling questions asked at the end of the resting state fMRI scan.

Dimension	Question:<br>My thoughts...
Vivid	... were vivid as if I was there
Normal	... were similar to thoughts I often have
Future	... involved future events
Negative	... were about something negative
Detailed	... were detailed and specific
Words	... were in the form of words
Evolving	... tended to evolve in a series of steps
Spontaneous	... were spontaneous
Positive	... were about something positive
Images	... were in the form of images
Other	... involved other people
Past	... involved past events
Deliberate	... were deliberate
Self	... involved myself
Stop	... were hard for me to stop
Distant time	... were related to a more distant time
Abstract	... were about ideas rather than events or objects
Decoupling	... dragged my attention away from the external world
Important	... were on topics that I care about
Intrusive	... were intrusive
Problem solving	... were about solutions to problems (or goals)
Here and now	... were related to the here and now
Creative	... gave me a new insight into something I have thought about before
Realistic	... were about an event that has happened or could take place
Thematic	... at different points in time were all on the same theme

Table S2. Component loadings and variance explained by each component from the principal component analysis with varimax rotation to the scores describing the participants' experience at the end of the resting state scan.

Dimension	Component 1	Component 2	Component 3	Component 4	Component 5	Component 6	Component 7	Component 8
Vivid	0.201	0.242	0.017	0.074	0.047	0.516	0.174	0.301
Normal	0.198	0.229	0.216	0.465	0.212	-0.235	0.073	-0.304
Future	-0.099	0.019	0.818	0.052	-0.168	-0.063	-0.09	0.131
Negative	0.211	0.053	0.138	-0.686	0.299	-0.157	0.114	-0.005
Detailed	0.15	0.501	0.088	0.192	0.258	0.074	0.008	0.091
Words	0.02	0.112	0.061	0.086	-0.063	-0.844	0.018	0.062
Evolving	0.175	0.332	-0.156	0.096	-0.147	-0.202	-0.312	-0.13
Spontaneous	0.347	-0.653	0.018	0.253	-0.111	-0.014	0.053	-0.144
Positive	-0.052	-0.107	0.111	0.775	0.099	0.089	-0.222	0.118
Images	0.199	-0.01	0.103	0.221	0.138	0.738	-0.055	-0.048
Other	0.166	-0.083	0.226	0.306	0.529	0.278	0.07	-0.01
Past	0.115	0.006	-0.137	-0.046	0.661	0.102	-0.123	-0.017
Deliberate	-0.136	0.704	-0.008	-0.046	-0.133	-0.058	0.064	-0.183
Self	0.01	-0.231	0.603	-0.017	0.194	0.189	0.222	-0.105
Stop	0.724	-0.158	0.049	-0.039	-0.041	0.062	0.112	-0.081
Distant∼time	0.116	-0.031	0.036	0.038	-0.011	-0.015	-0.038	0.844
Abstract	0.302	0.009	-0.064	0.072	-0.652	-0.021	0.059	0.028
Decoupling	0.402	0.031	0.033	0.245	-0.026	0.249	-0.147	0.138
Important	0.191	0.201	0.375	0.456	0.3	-0.03	0.158	-0.002
Intrusive	0.651	-0.073	-0.047	-0.146	0.179	0.046	0.014	0.11
Problem∼solving	0.343	0.358	0.528	-0.016	-0.189	0.034	-0.269	-0.153
Here∼and∼now	0.065	-0.094	-0.018	-0.201	-0.12	0.004	0.676	-0.205
Creative	0.444	0.292	0.135	0.07	-0.162	0.14	-0.158	0.233
Realistic	0.111	0.108	0.552	0.195	0.454	-0.034	-0.005	0.071
Thematic	-0.034	0.442	-0.013	0.019	-0.141	-0.053	0.6	0.258
Cumulative % of variance explained (after varimax rotation)	7.97	15.91	23.84	31.6	39.29	46.82	51.87	56.89

Table S3. Component loadings and variance explained by each component from the principal component analysis with varimax rotation to trait measures of well-being.

Dimension	Component 1	Component 2	Component 3
ADHD	-0.282	0.551	-0.024
Autism Spectrum Quotient	-0.181	-0.209	0.692
Depression	-0.711	0.4	0.229
Trait rumination	-0.461	0.638	0.235
State anxiety	-0.726	0.147	0.274
Trait anxiety	-0.643	0.44	0.468
Private Self Consciousness	-0.07	0.726	-0.192
Public Self Consciousness	0.008	0.748	0.31
Social anxiety	-0.06	0.247	0.774
Qualtiy of life and general health	0.711	-0.138	-0.017
Physical health	0.781	-0.195	0.02
Psychological health/well-being	0.606	-0.193	-0.418
Social relationships	0.636	0.014	-0.186
Environmental well-being	0.681	-0.06	0.016
Cumulative % of variance explained (after varimax rotation)	29.5	46.48	59.5

References

[1] Organization, W. H. *et al.* Who quality of life-bref (whoqol-bref). *Geneva: World Heal. Organ.* (2012).

[2] Scheier, M. & Carver, C. Self-consciousness scale–(scs-r). *Meas. Instrum. Database for Soc. Sci.* (2013).

[3] Spielberger, C. D. & Gorsuch, R. L. *Manual for the state-trait anxiety inventory (form Y):(" self-evaluation questionnaire")* (Consulting Psychologists Press, Incorporated, 1983).

[4] Treynor, W., Gonzalez, R. & Nolen-Hoeksema, S. Rumination reconsidered: A psychometric analysis. *Cogn. therapy research* **27**, 247–259 (2003).

[5] Radloff, L. S. The ces-d scale: A self-report depression scale for research in the general population. *Appl. psychological measurement* **1**, 385–401 (1977).

[6] Baron-Cohen, S., Wheelwright, S., Skinner, R., Martin, J. & Clubley, E. The autism-spectrum quotient (aq): Evidence from asperger syndrome/high-functioning autism, malesand females, scientists and mathematicians. *J. autism developmental disorders* **31**, 5–17 (2001).

[7] Kessler, R. C. *et al.* The world health organization adult adhd self-report scale (asrs): a short screening scale for use in the general population. *Psychol. medicine* **35**, 245–256 (2005).

[8] Munkres, J. Algorithms for the assignment and transportation problems. *J. society for industrial applied mathematics* **5**, 32–38 (1957).

[9] Margulies, D. S. *et al.* Situating the default-mode network along a principal gradient of macroscale cortical organization. *Proc. Natl. Acad. Sci.* 201608282 (2016).

[10] Yeo, B. T. *et al.* The organization of the human cerebral cortex estimated by intrinsic functional connectivity. *J. Neurophysiol.* **106**, 1125 (2011).

[11] Vidaurre, D., Smith, S. M. & Woolrich, M. W. Brain network dynamics are hierarchically organized in time. *Proc. Natl. Acad. Sci.* **114**, 12827–12832 (2017).
